# Extracellular vesicle-encapsulated microRNA-424 exerts inhibitory function in ovarian cancer by targeting MYB

**DOI:** 10.1186/s12967-020-02652-x

**Published:** 2021-01-06

**Authors:** Ping Li, Hongyan Xin, Lili Lu

**Affiliations:** 1 Department of Obstetrics and Gynecology, Linyi People’s Hospital, No. 27, Eastern Section of Jiefang Road, Lanshan District, Linyi, Shandong 276000 People’s Republic of China; 2 Department of Ultrasonography, Linyi People’s Hospital, Linyi, 276000 People’s Republic of China

**Keywords:** Ovarian cancer, Extracellular vesicle, Mesenchymal stem cell, microRNA-424, MYB, Human umbilical vein endothelial cell, Angiogenesis, VEGF

## Abstract

**Background:**

Recent studies have suggested a crucial role of mesenchymal stem cell (MSC)-derived extracellular vesicles (EVs) in ovarian cancer treatment. We, therefore, set out to explore the mechanism through which MSC-derived EVs delivered microRNA-424 (miR-424) to influence the development of ovarian cancer.

**Methods:**

Bioinformatics analyses were first performed to screen ovarian cancer-related differentially expressed genes and to predict regulatory miRNAs. Then, dual-luciferase reporter gene assay was carried out to verify the relationship between miR-424 and MYB. Subsequently, the characterized MSCs and isolated EVs were co-cultured with ovarian cancer cells, followed by determination of the expression patterns of miR-424, MYB, vascular endothelial growth factor (VEGF), and VEGF receptor (VEGFR), respectively. In addition, the effects of EVs-delivered miR-424 on the proliferation, migration, invasion and tube formation of ovarian cancer cells were assessed using gain- and loss-of-function approaches. Lastly, tumor xenograft was induced in nude mice to illustrate the influence of EVs-loaded miR-424 on ovarian cancer in vivo.

**Results:**

Our data exhibited that MYB was highly-expressed and miR-424 was poorly-expressed in ovarian cancer. More importantly, MYB was identified as a target gene of miR-424. Additionally, the transfer of miR-424 by MSC-derived EVs was found to repress the proliferation, migration, and invasion of ovarian cancer cells, with a reduction in the expressions of VEGF and VEGFR. Furthermore, MSC-derived EVs over-expressing miR-424 could inhibit the proliferation, migration, and tube formation of human umbilical vein endothelial cells, and also suppressed tumorigenesis and angiogenesis of ovarian tumors in vivo.

**Conclusion:**

Collectively, our findings indicate that MSC-derived EVs transfer miR-424 to down-regulate MYB, which ultimately led to the inhibition of the tumorigenesis and angiogenesis of ovarian cancer. Hence, this study offers a potential prognostic marker and a therapeutic target for ovarian cancer.

## Background

A heterogenous neoplasm, ovarian cancer is classified into various histological subtypes based on diverse recognizable original cells, molecular components, risk factors, clinical characteristics, and therapeutic methods [1, 2]. A large proportion of the females suffering from ovarian cancer at an advanced stage often present with disease relapse, accompanied by gradually shortened disease-free intervals [3]. Thus, it is imperative to develop novel and effective therapeutic approaches to combat the severity of this disease and improve the quality of life of women affected by the malignancy. Interestingly, mesenchymal stem cells (MSCs) have surfaced as promising candidates for treating various diseases, and MSCs hold significant importance in ovarian cancer therapeutics [4, 5]. In addition, the hard-done work of our peers has also highlighted the therapeutics importance of MSC-derived extracellular vesicles (EVs) [6], particularly the promising role of EVs in the diagnosis and treatment of ovarian cancer [7].

In the world of medicine, EVs are characterized as a group of cell-derived membrane structures including exosomes and microvesicles [8]. More importantly, EV-carrying microRNAs (miRNAs) possess the ability to serve as crucial factors by displaying a suppressive role in ovarian cancer [9]. It should be noted that EVs are capable of carrying proteins, messenger RNAs (mRNAs), and miRNAs, with an efficient ability to transfer from cell to cell [10]. Meanwhile, a specific miRNA, the miR-424 is known to suppress the cell migration and proliferation of ovarian cancer [11], and further confers a suppressive effect on malignant phenotypes in ovarian clear cells through the down-regulation of doublecortin-like kinase 1 [12]. Furthermore, miR-424 is classified as a large family cluster, which can inhibit the proliferation of tumor cells and promote cancer cell apoptosis in vivo and in vitro [13]. Poorly expressed miR-424 has been indicated to be correlated with clinical failure in prostate cancer [14]. In addition, overexpressed miR-424 presented suppressive effects in cell invasion and migration of cervical squamous cell carcinoma [15]. Therefore, it can be speculated that miR-424 may participate in the pathogenesis and development of ovarian cancer.

Additionally, the transcriptional factors MYB “featured with the presence of conserved DNA-binding domains (MYB domain)”, is known to participate in various processes like plant growth, stress responses, and metabolism [16]. Importantly, MYB has been suggested to function as a crucial factor in pathways related to the development of clear cell ovarian cancer [17]. Moreover, MYB has been previously demonstrated as the target gene of miR-150, while miR-150 has been reported to inhibit the melanoma cell development through the down-regulation of MYB [18]. Considering these above-mentioned findings, we anticipated that miR-424 from MSC-derived EVs possess a crucial role in ovarian cancer development via the regulation of MYB. We, therefore, set out to testify the hypothesis and to explore novel therapeutic biomarkers for ovarian cancer using a series of in vitro and in vivo experiments.

## Materials and methods

### Ethics statement

All patients provided signed informed consent prior to enrollment. The experiment protocols were conducted with approval of the Medical Ethics Committee of Linyi People’s Hospital and in accordance with *the Declaration of Helsinki*. The animal use and experimental procedures in this study were approved by the animal ethics committee of Linyi People’s Hospital.

### Microarray data analysis

Using the Gene Expression Omnibus (GEO) database (https://www.ncbi.nlm.nih.gov/geo/), we downloaded ovarian cancer-related microarray data GSE4122, GSE18520 and GSE38666 as well as the gene annotation files (Table 1). The limma package of R language was employed for differential analysis of the microarray data gene with the screening threshold of |log2FoldChange (FC)|> 2.0 and *adj.P.Val* < 0.05, with plotting of the heat map for differentially expressed genes (DEGs). The jvenn (http://jvenn.toulouse.inra.fr/app/example.html) was applied to compare the DEGs from three microarray data, the String database (https://string-db.org/) was employed to analyze the interaction of DEGs, and the Cytoscape 3.6.0 software [19] was used to construct the protein–protein interaction (PPI) of DEGs to screen the DEGs. To further explore the molecular mechanisms incorporating DEGs, a combination of the TarBase (http://carolina.imis.athena-innovation.gr/diana_tools/web/index.php?r=tarbasev8%2Findex), miRDB (http://www.mirdb.org/), mirDIP (http://ophid.utoronto.ca/mirDIP/), miRTarBase (http://mirtarbase.mbc.nctu.edu.tw/php/search.php), TargetScan (http://www.targetscan.org/vert_71/) and miRWalk (http://mirwalk.umm.uni-heidelberg.de/) were employed to predict the regulatory miRNAs of DEGs, and the predicted results were compared using jvenn.

**Table 1:** The information of ovarian cancer-related microarray data

Accession	Platform	Organism	Sample
GSE4122	GPL201	Homo sapiens	14 normal human ovary specimens and 32 malignant human ovary specimens
GSE18520	GPL570	Homo sapiens	53 papillary serous ovarian adenocarcinomas specimens and 10 normal ovarian surface epithelium specimens
GSE38666	GPL570	Homo sapiens	8 ovarian normal stroma specimens and 7 ovarian cancer stroma specimens

### Sample collection

The ovarian tumor samples were harvested from 85 patients following initial diagnosis as ovarian cancer at the Linyi People’s Hospital from July 2014 to April 2016. All enrolled patients were aged between 36 and 83 years with an average age of 59.68 ± 13.72 years, without prior history of radiotherapy or chemotherapy. Moreover, in our study, 35 cases were classified in an early stage while 50 cases were in the advanced stage according to the International Federation of Gynecology and Obstetrics (FIGO) staging classification (2013). All samples were harvested by surgical intervention and the patients were followed up for 3 years. Additional 43 cases of adjacent ovarian epithelium tissues were harvested for this study.

### Reverse transcription-quantitative polymerase chain reaction (RT-qPCR)

The total RNA content was extracted using the Trizol Reagent (Invitrogen, Carlsbad, CA, USA) and integrity of the total RNA content was evaluated by gel electrophoresis. Successful isolation of total RNA content was indicated by clear appearance of the 28S band and 18S band in electrophoretogram, wherein the 28S band was twice the length of 18S. Next, Nanodrop2000 (Thermo Fisher Scientific, Waltham, USA) was used to detect the absorbance at excitation wavelengths of 260 and 280 nm for determining the RNA concentration, and 1 µg total RNA was reversely transcribed into complementary DNA (cDNA) using PrimeScript™ RT reagent kit with the gDNA Eraser kit (RRO37A, Takara Bio Inc., Otsu, Shiga, Japan). Then, RT-qPCR was conducted using the SYBR®*Premix Ex* Taq™ (Tli RNaseH Plus) kit (RR820A, Takara) in the ABI7500 quantitative PCR instrument (Thermo Fisher Scientific). Additionally, the synthetic *C. Elegans* (cel)-miR-39 was used as a spike-in control for the total RNA content extracted from EVs. The relative mRNA and miRNA expression, normalized to glyceraldehyde-3-phosphate dehydrogenase (GAPDH) and U6 respectively, was determined based on the 2^−ΔΔCt^ method. All primers were provided by GenePharma Co., Ltd., (Shanghai, China) and the primer sequences are shown in Table 2.

**Table 2:** Primer sequences for RT-qPCR

Gene	Primer sequence (5′-3′)
miR-424	Forward: GGCAGCAGCAATTCATG
	Reverse: CAGTGCGTGTCGTGGAGT
MYB	Forward: AGAAACGAGCCTGCCTGCCTTACA
	Reverse: AGATGGTTCCTCAGGGAGGT
cel-miR-39	UCACCGGGUGUAAAUCAGCUUG
U6	Forward: CAGGGGCCATGCTAAATCTTC
	Reverse: CTTCGGCAGCACATATACTAAAAT
GAPDH	Forward: CTCATGACCACAGTCCATGCCA
	Reverse: GGATGACCTTGCCCACAGCCTT

*RT-qPCR* reverse transcription quantitative polymerase chain reaction, *miR* microRNA, *GAPDH* glyceraldehyde-3-phosphate dehydrogenase

### Western blot analysis

Phenylmethanesulfonyl fluoride and protease inhibitor (P0100, Beijing Solarbio Science & Technology Co., Ltd., Beijing, China) were added into the Radio Immunoprecipitation Assay lysis to extract total protein content from the tissues and cells. Total proteins were lysed at 4 °C for 15 min and centrifuged at 15,000 rpm for 15 min to isolate the supernatant followed by determination of protein concentration using the bicinchonininc acid kit (23227, Thermo Fisher Scientific). After protein quantitation, the proteins were separated by polyacrylamide gel electrophoresis and transferred onto polyvinylidene fluoride membranes. Thereafter, the membrane was blocked with 5% bovine serum albumin for 1 h at room temperature followed by overnight incubation at 4 °C with the corresponding primary antibodies MYB (dilution ratio of 1: 2500, ab12296, Abcam Inc., Cambridge, UK), vascular endothelial growth factor (VEGF; dilution ratio of 1: 100, AF-293-NA, R&D Systems, Minneapolis, MN, USA), vascular endothelial growth factor receptor (VEGFR; dilution ratio of 1: 100, ab11939, Abcam) and GAPDH (dilution ratio of 1: 5000, ab8245, Abcam). The following day, the membranes were washed with Tris-buffered saline with 0.1% Tween-20 (TBST) 3 times (5 min for each), incubated with horseradish peroxidase (HRP)-labeled goat anti-rabbit immunoglobulin G (IgG; dilution ratio of 1: 20,000, ab205718, Abcam) for 1.5 h at room temperature, and rinsed 3 times (5 min for each) with TBST again. Subsequently, the membranes were developed with the addition of the developer (NCI4106, Pierce, Rockford, IL, USA), followed by quantitative protein analysis using the ImageJ 1.48u software (Bio-Rad, Hercules, CA, USA). The relative expression of proteins was expressed as the ratio of the gray value of proteins to that of GAPDH.

### Immunohistochemistry

The tissue samples were made into paraffin sections, hydrated with gradient alcohol, washed under running water for 2 min, with 3% methanol H_2_O_2_ for 20 min, with distilled water for 2 min, and rinsed with 0.1 M phosphate-buffered saline (PBS) for 3 min. Thereafter, the sections were incubated with normal goat serum sealing solution (C-0005, Shanghai Haoran Bio Technologies Co., Ltd. Shanghai, China) at room temperature for 20 min, and with the primary antibody rabbit anti-human MYB (dilution ratio of 1: 100, ab76009, Abcam) at 4 °C overnight and rinsed with 0.1 M PBS thrice (5 min for each). Next, the sections were added with the secondary antibody goat anti-rabbit IgG (ab6785, dilution ratio of 1: 1000, Abcam) at 37 °C for 2 min. Subsequently, the sections were incubated with HRP-labeled streptavidin ovalbumin working solution (0343-10000U, Imunbio, Beijing, China) at 37 °C for 20 min. The sections were then developed with diaminobenzidine (ST033, Whiga, Guangzhou, China), counterstained by hematoxylin (PT001, Shanghai Bogoo Biotechnology, Co., Ltd., Shanghai, China) for 1 min, added with 1% ammonia water to return to blue, dehydrated with gradient alcohol, cleaned by xylene, and mounted by neutral balsam. Bright field images were acquired using a Nikon Eclipse TE2000 microscope (Nikon, Tokyo, Japan) and NIS-Elements software with four pictures taken from each experiment. Analysis and quantification on images were performed using the ImageJ software.

### Cell culture and transfection

The normal human ovarian epithelial cell line HOSEpiC and human ovarian cancer cell lines (SKOV-3, HO8910, A2780) and human umbilical vein endothelial cells (HUVECs) were purchased from the Shanghai Institute of Cellular Biology of Chinese Academy of Sciences (Shanghai, China). The procured cells were cultured with Roswell Park Memorial Institute (RPMI) 1640 medium (Gibco by Life Technologies, Grand Island, NY, USA) containing 10% fetal bovine serum (FBS) and penicillin–streptomycin (Gibco by Life technologies) and incubated at 37 °C with 5% CO_2_. Following incubation, the cells were detached with 0.25% trypsin, sub-cultured at the ratio of 1: 3, and seeded into 6-well plates at a density of 3 × 10^5^ cells/well. After achieving 70–80% cell confluence, cells at the logarithmic phase of growth were selected for subsequent experiments.

The ovarian cancer cells at logarithmic phase of growth were seeded in 6-well plates at a density of 4 × 10^5^ cells/well. When cell confluence reached 80–90%, the cells were transfected based on the instructions of lipofectamine 2000 (11668-019, Invitrogen). Following transfection, the cells were cultured with 5% CO_2_ at 37 °C under the condition of saturated humidity. After 48 h, the original medium in wells was discarded, and the cells were added with RPMI 1640 medium containing 10% FBS for another 24–48 h. Next, the cells were grouped by transfecting with mimic-negative control (mimic-NC), miR-424 mimic, inhibitor-NC, miR-424 inhibitor, NC for short hairpin RNA (sh-NC), and sh-MYB, alone or in combination. All the aforementioned transfection sequences and plasmids were purchased from GenePharma.

### Isolation and culture of MSCs and osteogenic/adipogenic identification

Healthy and well-grown C57BL/6 mice were collected, euthanized and soaked into alcohol for 10 min. Under aseptic conditions, the femur and tibia of mice were collected with the leg flesh removed, and then washed and placed in a flat dish containing Dulbecco’s modified Eagle medium (DMEM) (Gibco by Life Technologies) for further use. After that, both ends of the femur and tibia were cut open to isolate the bone marrow cells which were then mixed with DMEM into a 15 mL centrifuge tube using injector followed by centrifugation at 1500 rpm for 3 min, with the supernatant discarded. Subsequently, freshly isolated MSCs were resuspended by DMEM containing 10% FBS (Biowest, Nuaillé, France) supplemented with 100 U/mL penicillin–streptomycin (Gibco by Life Technologies) and cultured with 5% CO_2_ at 37 °C and the medium was refreshed after 3 days. Thereafter non-adherent cells were removed and cell morphology of MSCs was observed, photographed, and recorded. After attaining 80–90% confluence, the cells were subsequently sub-cultured in different bottles while cells at passage 3 (P3) were collected for further experimentation.

The MSCs at P3 were collected, detached with trypsin (Gibco by Life Technologies), resuspended, and counted while cell concentration was adjusted to 1 × 10^6^ cells/mL. Following after, 200 μL of cell suspension was collected and placed into an Eppendorf (EP) tube, mixed with 5 μL different fluorescent-labeled monoclonal antibodies (CD19, CD34, CD45, CD73, CD90, CD105, and HLA-DR), and then incubated at 4 °C for 15 min avoiding exposure to light. After that, cells in each tube were added with 2 mL PBS, triturated, mixed, and centrifuged at 1000 rpm for 5 min, with the supernatant discarded to remove uncombined antibodies. After that, the cells were resuspended with 0.01 M PBS (400 μL) containing 0.5% paraformaldehyde and mixed uniformly. The IgG labeled with the same fluorescence was set as the isotype control while a flow cytometer (BD Biosciences, San Jose, CA, USA) was used to characterize the cells.

The MSCs at P3 were trypsinized and made into a single-cell suspension, with the cell concentration adjusted to 1 × 10^5^ cells/mL. Cells in the experimental group were added with 2 mL osteogenic differentiation induction complete medium (MUBMX-90021, Cyagen Biosciences Inc., Guangzhou, China), while the cells in control group were added with equal amounts of DMEM. After 3 days of cell culture, medium in both the experimental group and control group was replaced with a fresh medium. After 2–3 weeks of induction, the medium was removed and cells were rinsed twice with PBS. Cells in each well were added with 2 mL 4% neutral formaldehyde to fix for 30 min, with formaldehyde then removed. After another two rinses with PBS, cells in each well were stained with 1.5 mL alizarin red for 5 min. Lastly, the cells were rinsed with PBS twice, observed, and photographed under a microscope (TE2000, Nikon, Tokyo, Japan).

The MSCs at P3 were trypsinized and made into a single-cell suspension, with cell concentration adjusted to 3 × 10^5^ cells/mL. When cell confluence reached 80–90%, the supernatant was removed, and the cells were added with 2 mL of adipogenic differentiation medium A (MUBMX-90031, Cyagen Biosciences Inc., Guangzhou, China). After 3 days of induction, medium A in 6-well plate was removed, and the cells were added with 2 mL of adipogenic differentiation medium B. After 24 h of induction, medium B was replaced with medium A to induce cells, while alternate induction was conducted 5 times and then cells were induced with medium B for 7 days, with medium renewed every 3 days. After that, medium B in 6-well plate was removed and cells in each well were fixed with 2 mL of 4% neutral formaldehyde for 30 min. Next, the cells in each well were stained with 1.5 mL oil red O for 30 min. Finally, the cells were observed and photographed under a microscope (TE2000, Nikon, Tokyo, Japan).

### Extraction and identification of MSC-derived EVs

The MSCs at P3 were collected and cultured overnight in serum-free DMEM. Upon reaching 80–90% confluence, the cells were sub-cultured in serum-free medium for 24 h with supernatant collected. Thereafter, the cells were centrifuged at 2000 × *g* for 20 min at 4 °C to remove cell debris, and the collected supernatant was further exposed to high-speed centrifugation (100,000 × g) for 1 h at 4 °C. Following after, the cells were suspended in serum-free DMDM containing 25 mM HEPES (pH = 7.4), precipitated, and followed by high-speed centrifugation once again. After the removal of the supernatant, the cell precipitates were stored at − 80 °C for subsequent use.

Transmission electron microscopy (TEM) was applied to identify the EVs as follows: 30 μL EVs were placed on a copper mesh and allowed to stand for 1 min, with liquid dried using a filter paper. Next, the EVs were counterstained with 30 μL phosphotungstic acid solution (pH 6.8) for 5 min at room temperature, dried using a filament lamp and photographed by means of TEM.

Flow cytometry was further employed to detect the content of EVs surface marker CD63 as follows: the MSCs with good growth conditions were collected and rinsed with PBS. After detachment with trypsin, the cells were centrifuged at 1000 rpm for 5 min and the supernatant was discarded. Next, the cells were added with PBS and triturated into a single cell suspension. The cells were then evenly divided into 10 EP tubes (1.5 mL), centrifuged at 1000 rpm for 5 min, with the supernatant discarded. Following after, the cells were added with 1 mL PBS (containing 1% bovine serum albumin [BSA]) to disperse the precipitates, incubated at room temperature for 30 min to block nonspecific antigen, and centrifuged at 1000 rpm for 5 min with the supernatant discarded followed by resuspension with PBS (200 µL/EP tube). Subsequently, the cells were added with CD63-PE antibody and incubated at room temperature for 30 min, with the cells without antibody used as blank control and the PE-labeled anti-human IgG as an isotype control. After that, the cells were centrifuged at 1000 rpm for 5 min with supernatant discarded, resuspended with PBS containing 1% BSA, precipitated, and sampled followed by detection using a Guava easyCyte™ system flow cytometer. Total RNA content of miR-424 from isolated EVs was extracted using a Trizol reagent (Invitrogen), and the isolated miR-424 was identified by means of agarose gel electrophoresis (Additional file 1: Fig. S1). The expression patterns of miR-424 in EVs were detected using miRcute miRNA qPCR Detection kits (SYBR Green; TIANGEN, Beijing, China).

### Dual-luciferase reporter gene assay

The plasmids PmirGLO-MYB-wild type (WT) and PmirGLO-MYB-mutant (MUT) were constructed and co-transfected with miR-424 mimic and mimic NC respectively into 293 T cells. After 24 h of transfection, the cells were lysed and centrifuged at 12,000 rpm for 1 min with supernatant collected. The luciferase activity was detected using a Dual-Luciferase® Reporter Assay System (E1910, Promega Corp., Madison, Wisconsin, USA), and the luciferase activity = firefly luciferase activity/Renilla luciferase activity.

### Co-culture of MSCs/EVs with ovarian cancer cells

To inhibit the secretion of MSC-derived EVs, MSCs were treated with EV release inhibitor GW4869. MSCs were then spread in a 6-well plate at a density of 1 × 10^6^ cells/well. After achieving 80–90% confluence, the cells were treated with 10% GW4869 (D1692-5MG, Sigma-Aldrich, St. Louis, MO, USA), whereas the controls were treated with 0.005% dimethyl sulphoxide.

Next, the MSCs were spread in the basolateral chamber of 24-well Transwell chamber at a density of 1 × 10^4^ cells/well, while the ovarian cancer cells were spread in the apical chamber. After 24 h of co-culture, the ovarian cancer cells were isolated to detect the expressions of miR-424 and MYB in cells. The MSCs used for detection were categorized as MSCs without treatment, EVs-depleted MSCs, and MSCs treated with miR-424 mimic or miR-424 inhibitor as well as their NCs. Thereafter, the secretion of MSC-derived EVs treated with GW4869 was observed under TEM.

In accordance with the instructions of PKH67 (Green) dye kits (PKH67GL-1KT, Sigma-Aldrich), MSC-derived EVs were labeled, and then the fluorescence-labeled EVs were co-cultured with the ovarian cancer cell (with 50–60% confluence) supernatant in a 24-well plate followed by 48 h of co-culture. The MSC-derived EVs used for detection were categorized as MSC-derived EVs without treatment, EVs-depleted MSCs, MSC-derived EVs treated with miR-424 mimic or miR-424 inhibitor as well as their NCs. The ovarian cancer cell supernatant was collected and the expressions of miR-424 and the mRNA and protein expressions of MYB were detected.

### 5-ethynyl-2′-deoxyuridine (EdU) assay

The ovarian cancer cells were isolated, rinsed twice with PBS, and seeded in a 96-well plate at a density of 5 × 10^3^ cells/well. After 6 h, the cells were added with EdU medium (100 μL/well) and incubated for 2 h. Next, the cells were incubated with cell fixation fluid (4% paraformaldehyde) (100 μL/well) at room temperature for 30 min, followed by incubation with 2 mg/mL glycine for 5 min and then a penetrant (PBS containing 0.5% TritonX-100) (100 μL/well) for 10 min. Afterwards, the cells were incubated with 1 × Apollo staining reaction solution (100 μL/well) for 30 min in dark conditions. Following after, the cells were incubated with 1 × Hoechst 33342 reaction solution (100 μL/well) on a decoloring shaking table at room temperature for 30 min avoiding exposure to light. After staining, the cells were added with an anti-fluorescence quenching sealer (100 μL/well). Under a fluorescence microscope (TE2000, Nikon, Tokyo, Japan), the cells were photographed and the number of EdU-labeled cells was recorded. The cells with red-stained nucleus were regarded as positive cells, whereas the positive and negative cells were counted within 3 random visual fields. The EdU labeling rate (%) = the number of positive cells/(the number of positive cells + the number of negative cells) × 100%.

### Transwell assay

A Transwell chamber (diameter = 8 mm; Corning, Corning Glass Works, Corning, N.Y., USA) was used for the detection of cell migration and invasion in a 24-well plate. In the polycarbonate membrane Transwell chamber with or without Matrigel, 600 ml of RPMI 1640 medium containing 20% FBS was added to the basolateral chamber and then balanced for 1 h at 37 °C. After 48 h of transfection, ovarian cancer cells were resuspended in FBS-free RPMI 1640 medium while 1 × 10^6^/mL cells were seeded in the apical chamber and cultured with 5% CO_2_ for 24 h at 37 °C. Subsequently, the Transwell chamber was fixed with 5% glutaraldehyde at 4 °C, and stained with 0.1% crystal violet for 5 min. Lastly, the cells were observed under an inverted fluorescence microscope (TE2000, Nikon, Tokyo, Japan) and photographed in 5 randomly-selected visual fields and the average value considered as the number of cells passed through the chamber.

### HUVEC treatment

HUVECs were cultured in a 24-well plate at a density of 1 × 10^5^ cells/well, followed by treatment with ovarian cancer cell supernatant co-cultured with MSC-derived EVs. After 24 h of treatment, HUVECs were isolated. Next, the HUVECs were respectively treated with MSC-derived EVs, EVs-depleted MSCs, and MSC-derived EVs carrying miR-424 mimic or miR-424 inhibitor as well as their NCs. Subsequently, the expression patterns of VEGF and VEGFR were determined using RT-qPCR and Western blot analysis.

### Matrigel-based tube formation assay

Matrigel (356,234, Shanghai Shanran Biotechnology Co., Ltd., Hangzhou, China) was placed in a refrigerator at 4 °C overnight to thaw into a yellow-colloidal liquid. Next, 70 μL of this yellow-colloidal liquid (0.5 mmol/L in thickness) was collected using pre-cooled pipettes and immediately added to a pre-cooled 96-well plate. Afterwards, the cell culture plates with Matrigel were placed in an incubator for 30 min at 37 °C for solidification. After 48 h of transfection, cells with different treatments were collected, and then treated with serum-free starvation for 1 h and resuspended in DMEM to make a cell suspension. Thereafter, the cell suspension (1 × 10^5^ cells/mL) was seeded in culture wells and added with the corresponding medium, and 3 replicates were set for each group. Subsequently, the cell culture plate was placed in an incubator for 18 h and photographed under a Leica inverted phase-contrast microscope (Leica Microsystems GmbH, Wetzlar, Germany). The Image-Pro Plus software (version 6.0) was employed to calculate the number of a complete capillary tube surrounded by cells, with at least 3 visual fields counted.

### Tumor xenograft in nude mice

A total of 60 immunodeficient female nude mice (aged 3—5 weeks, weighing 18–21 g) were procured from the Shanghai laboratory animal center, Chinese Academy of Sciences (Shanghai, China). The obtained mice were maintained under specific pathogen-free conditions with regular ultraviolet radiation. The cage, padding, water, and feed for mice were sterilized and the mice were kept at the temperature of 24–26 °C with a relative humidity of 40–60%. Subsequently, the cultured SKOV-3 cells were transfected with lv-NC (lentivirus over-expressing miR-424 NC) and lv-miR-424 mimic (lentivirus over-expressing miR-424) to obtain stably-transfected cell lines. The lentivirus vector and reagents used for aforementioned transfection were purchased from GenePharma. In addition, the cells were also treated with EVs-mimic NC and EVs-miR-424 mimics.

The stably-expressed ovarian cancer cells at the logarithmic phase of growth were collected, and PBS was used to adjust the concentration of cell suspension to 5 × 10^6^ cells/mL. One week later, the cells treated with lv-NC and lv-miR-424 mimic were injected into the mice (n = 15) via the abdomen (every 0.2 mL cell suspension contained 1 × 10^6^ cells), with 200 μL cell suspension injected every day. Meanwhile, the cells treated with EVs-mimic NC and EVs-miR-424 mimic (60 μg/mL) were injected into mice (n = 15) via the tail vein. After injection for 4 consecutive weeks, all mice were euthanized and dissected, and the tumors were removed. By accurately measuring the short diameter (a) and long diameter (b) of tumors, the tumor volume was calculated based on the following formula: π(a^2^b)/6, and the tumors were also weighed. Additionally, when the mice were euthanized, peripheral blood samples were collected to prepare serum, and then serum EVs were extracted to detect the miR-424 expressions using RT-qPCR. Tumor tissues were fixed by 10% formaldehyde, conventionally dehydrated, paraffin-embedded, and cut into 4-μm sections for subsequent use. The protein expression patterns of MYB and VEGF were detected by Western blot analysis.

### Measurement of microvessel density (MVD)

The MVD in tumor tissues of nude mice was measured using the detection method of immunohistochemistry and the primary antibody rabbit anti-mouse MVD was purchased from Santa Cruz Biotechnology (sc-376975). The criteria for MVD results were as follows: using the Weidner microvessel count method, in endometrial tissues, any brown-yellow stained cells or cell clusters with clear boundary with adjacent microvasculature, and gland tissues were considered as a new blood vessel, while the branches were also regarded as a blood vessel as long as the structure was not connected. However, the formation of tube structure was not necessary for analysis, and therefore we did not count the cells with a large tube and a thick muscular layer or with more than 8 red blood cells in the tube. Meanwhile, the brown-yellow stained vessels were selected and counted with 5 visual fields were randomly selected for each pathological section to calculate the average value, which was then used as MVD. Moreover, MVD was considered as positive when the average value was ≥ MVD threshold and as negative when the average value was < MVD threshold (MVD threshold was set based on the Weidner criteria) [20].

### Statistical analysis

Statistical analyses were performed using the SPSS 21.0 statistical software (IBM Corp., Armonk, NY, USA). Measurement data were presented as mean ± standard deviation. Comparisons between tumor tissues and adjacent tissues were conducted using a paired *t*-test, and comparisons between other two groups were performed using an independent sample *t*-test. Data among multiple groups were compared by one-way analysis of variance (ANOVA), and Tukey’s was used for the post-hoc test. Data among groups at different time points were compared by repeated-measures ANOVA, with Bonferroni used for post-hoc test. Kaplan–Meier survival analysis was employed to analyze the relationship between MYB expression and ovarian cancer survival rate, and the Log-rank method was used for differential analysis. Pearson correlation analysis was used to analyze the correlation between miR-424 and MYB. A value of *p* < 0.05 was considered statistically significant.

## Results

### The significance of MYB and miR-424 in ovarian cancer

**Fig. 1:**
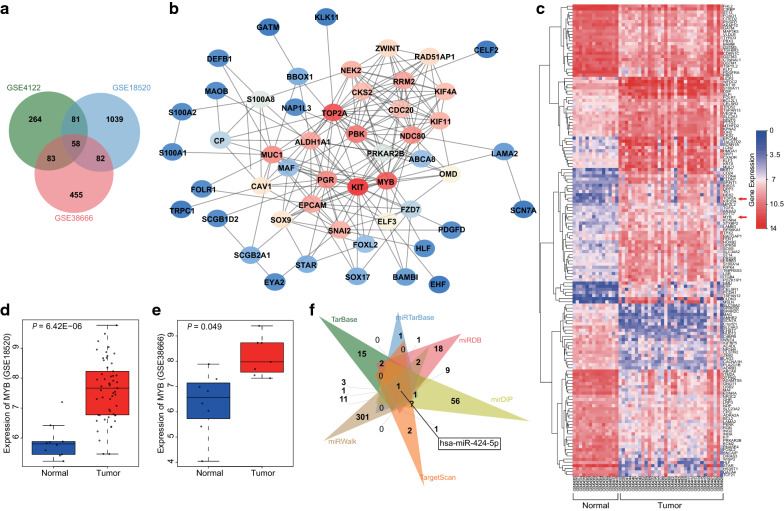
MYB is upregulated in ovarian cancer and is predicted to be mediated by miR-424. **a** Comparison among DEGs from ovarian cancer-related microarray data GSE4122, GSE18520 and GSE38666, suggesting 59 intersected genes. **b** The PPI network of 58 intersected genes. **c** The heat map for the expression of the first 150 DEGs in GSE4122; the abscissa indicates sample number and the ordinate indicates DEGs, and histogram in the upper right indicates color gradation, and each rectangle indicates one corresponding sample expression value. **d** The expression of MYB in GSE18520. **e** The expression of MYB in GSE38666. **f** The regulatory miRNAs of MYB predicted using TarBase, miRDB, mirDIP, miRTarBase, TargetScan, and miRWalk

Firstly, differential analyses for microarray data GSE4122, GSE18520 (Additional file 2: Fig. S2a) and GSE38666 (Additional file 2: Fig. S2b) were performed using the R language with the screening threshold of |log2FC|> 2.0 and *adj.P.Val* < 0.05, which revealed 486, 1260 and 678 DEGs, respectively. Subsequently, a Venn diagram was plotted (Fig. 1a) and 58 genes were found at the intersection. The obtained 58 intersected genes were then subjected to analysis with the String database to identify intersection among genes, and the results demonstrated that there were 6 genes not interacting with the other genes. As a result, the remaining 52 genes were further analyzed to establish a PPI network (Fig. 1b). Results from the PPI network revealed that the KIT, TOP2A, and MYB genes exhibited a higher correlation with other genes, and were thus likely to affect the development of ovarian cancer. Moreover, the heat map for the expression patterns of the first 150 DEGs in GSE4122 was plotted (Fig. 1c), which revealed that TOP2A and MYB were highly-expressed in ovarian cancer. Various studies have shown the association between TOP2A and ovarian cancer [21, 22]; however, in-depth investigations about the mechanism of MYB in ovarian cancer remain scarce. We, therefore, focused on the role of MYB on ovarian cancer. The expression patterns of MYB in microarray data GSE18520 and GSE38666 is shown in Fig. 1d, e, and illustrated higher expression levels of MYB in ovarian cancer tissues relative to normal tissues. In addition, the regulatory miRNAs of MYB were predicted using the TarBase, miRDB, mirDIP, miRTarBase, TargetScan, and miRWalk databases. Finally, a total of 36, 46, and 114 miRNAs were indicated by TarBase, miRDB, and mirDIP, respectively. Moreover, 11 miRNAs were predicted with “Strong evidence” from miRTarBase, and the binding site was located in the 3′untranslated regions (3′UTRs) as stated by TargetScan and miRWalk, whereas there were 330 miRNAs with energy < − 17 predicted from miRWalk, while 26 miRNA with Pct > 0.5 were predicted from TargetScan. By comparing the prediction results from the aforementioned 6 databases (Fig. 1f), it was found that there was only one intersected miRNA of has-miR-424-5p, suggesting that miR-424 was likely to regulate MYB. Therefore, we speculated that miR-424 affected ovarian cancer by targeting MYB.

### miR-424 is poorly-expressed in ovarian cancer and targets MYB

**Fig. 2:**
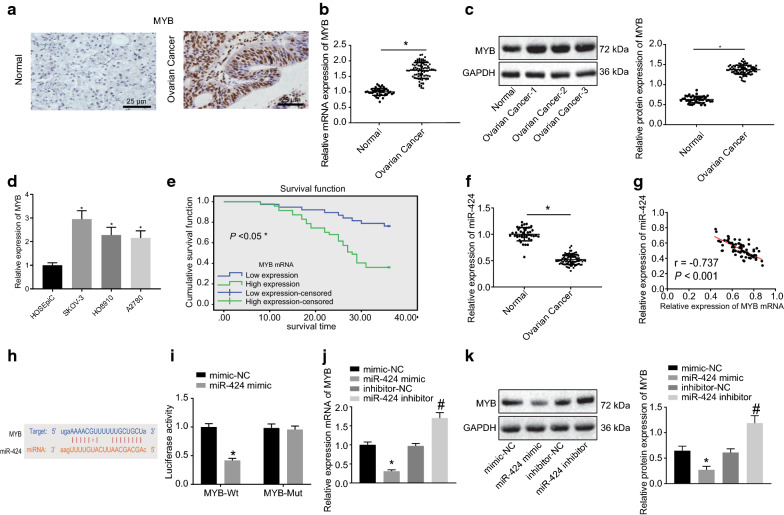
miR-424 is downregulated in ovarian cancer and specifically binds to MYB. **a** The expression of MYB in ovarian cancer tissues and adjacent ovarian epithelium tissues examined by immunohistochemistry (scale bar = 25 μm), adjacent ovarian epithelium tissues n = 43, ovarian cancer tissues n = 85. **b** The mRNA expression of MYB in ovarian cancer tissues and adjacent ovarian epithelium tissues detected by RT-qPCR; *p < 0.05. **c** The protein expression of MYB in ovarian cancer tissues and adjacent ovarian epithelium tissues examined by Western blot analysis. **d** The expression of MYB in normal human ovarian epithelial cell line HOSEpiC and ovarian cancer cell lines (SKOV-3, HO8910, A2780) detected by RT-qPCR. **e** The survival rate of ovarian cancer patients with higher MYB expression analyzed by Kaplan–Meier survival analysis. **f** The expression of miR-424 in ovarian cancer tissues and adjacent ovarian epithelium tissues detected by RT-qPCR; *p < 0.05. **g** The correlation between MYB and miR-424. **h** The binding site between miR-424 and MYB predicted using an online data base. **i** The fluorescence activity determined by dual-luciferase reporter gene assay. **j** The mRNA expression of MYB in cells after treatments with miR-424 mimic or inhibitor detected by RT-qPCR. **k** The protein expression of MYB in cells after treatments with miR-424 mimic or inhibitor examined by Western blot analysis; *p < 0.05 compared with cells treated with mimic-NC, ^#^*p* < 0.05 compared with cells treated with inhibitor-NC. All data above were measurement data and were presented as mean ± standard deviation. A comparison between tumor tissues and adjacent ovarian epithelium tissues was conducted using a paired *t*-test, and data comparison between two groups was performed using the unpaired *t*-test. Data among multiple groups were compared by one-way ANOVA, and Pearson correlation analysis was used to analyze the correlation between miR-424 and MYB. All experiments were repeated 3 times independently

Immunohistochemistry was employed to detect the positive expressions of MYB in ovarian cancer, and it was found that the positive expression of MYB was brown-yellow in color and located in the nucleus (Fig. 2a). In comparison with the adjacent ovarian epithelium tissues, ovarian cancer tissues presented with higher positive expression of MYB (*p* < 0.05) (Fig. 2b, c). Next, RT-qPCR was conducted to examine the expression patterns of MYB in normal human ovarian epithelial cell line HOSEpiC and ovarian cancer cell lines (SKOV-3, HO8910, and A2780), which revealed higher expression of MYB in SKOV-3, HO8910, and A2780 compared to that in HOSEpiC, and demonstrated the largest FC in SKOV-3 (*p* < 0.05) (Fig. 2d). These results demonstrated that MYB was highly-expressed in ovarian cancer tissues and cells. Meanwhile, Kaplan–Meier survival analysis (http://kmplot.com/analysis/index.php?p=service&cancer=gastric) (Fig. 2e) indicated that the ovarian cancer patients presenting with higher MYB expression had a lower overall survival rate compared to those with lower MYB expression levels.

RT-qPCR was further performed to detect the expression patterns of miR-424 in ovarian cancer tissues, and miR-424 was found to be significantly down-regulated in ovarian cancer tissues relative to adjacent ovarian epithelium tissues (Fig. 2f). By correlation analysis, a negative correlation between MYB and miR-424 was found (Fig. 2g). Further application of the online analysis software indicated the presence of a specific binding region between the MYB gene sequence and miR-424 sequence (Fig. 2h). Subsequently, dual-luciferase reporter gene assay verified that MYB was indeed the target of miR-424. As shown in Fig. 2i, the fluorescence activity was significantly reduced after co-transfection of miR-424 mimic and MYB 3′UTR-WT compared with co-transfection of mimic-NC and MYB 3′UTR-WT (*p* < 0.05), while the fluorescence activity exhibited no pronounced differences following co-transfection of miR-424 mimic and MYB 3′UTR-MUT in comparison with the co-transfection of mimic-NC and MYB 3′UTR-WT (*p* > 0.05), suggesting that miR-424 specifically bound to MYB. Furthermore, SKOV-3 cells were treated with miR-424 mimic or inhibitor followed by RT-qPCR and Western blot analysis to examine the MYB expression in SKOV-3 cells. It was found that in comparison with cells treated with mimic-NC, the cells treated with miR-424 mimic presented with significantly reduced mRNA expressions of MYB (Fig. 2j) and protein expressions of MYB (Fig. 2k), while the cells treated with miR-424 inhibitor exhibited markedly increased mRNA expressions of MYB (Fig. 2j) and protein expressions of MYB (Fig. 2k) relative to the cells treated with inhibitor-NC (all *p* < 0.05). The above-reported results suggested that miR-424 regulated the expression of MYB, and augmented miR-424 suppressed the MYB expression.

### miR-424 inhibits the cell proliferation, migration and invasion of ovarian cancer by suppressing MYB

**Fig. 3:**
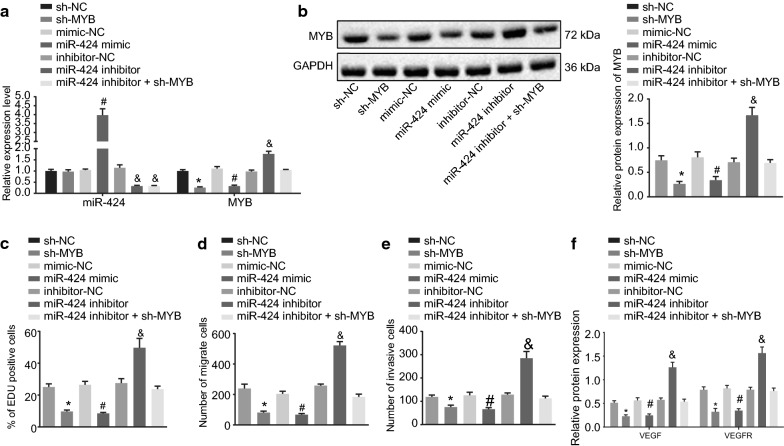
The proliferation, migration, and invasion of ovarian cancer cells are repressed by miR-424 via inhibition of MYB. The cells used for the following detections were treated with sh-NC, sh-MYB, miR-424 mimic or inhibitor as well as their NCs, and both miR-424 inhibitor and sh-MYB. **a** The expression of miR-424 and MYB in SKOV-3 cells after different treatments detected by RT-qPCR. **b** The protein expression of MYB in cells after different treatments examined by Western blot analysis. **c** The proliferation of SKOV-3 cells after different treatments determined by EdU assay. **d** The migration of SKOV-3 cells after different treatments detected by Transwell assay. **e** The invasion of SKOV-3 cells after different treatments examined by Transwell assay. **f** The protein expression of VEGF and VEGFR in cells after different treatments detected by Western blot analysis, with protein bands assessed; *p < 0.05 compared with cells treated with mimic-NC, ^#^*p* < 0.05 compared with cells treated with inhibitor-NC; ^&^*p* < 0.05 compared with cells treated with inhibitor NC. The data above were measurement data and were presented as sample mean ± standard deviation. Data among multiple groups were compared by one-way ANOVA, with Tukey’s post hoc test conducted. Data in each group at different time points were compared by repeated-measures one-way ANOVA. The cell experiment was repeated 3 times independently

Subsequently, SKOV-3 cells were treated with interfered MYB to examine changes in MYB expression. As depicted in Fig. 3a, b, the sh-MYB treatment led to significantly reduced expressions of MYB in ovarian cancer cells, while miR-424 inhibitor transfection brought about the opposite results. In addition, EdU assay was applied to detect the proliferation ability of SKOV-3 cells (Fig. 3c), which revealed that cells delivered with miR-424 inhibitor presented with markedly increased red-positive proliferation, while the cells treated with both miR-424 mimic and sh-MYB showed a significant decrease in red-positive proliferation (*p* < 0.05). Meanwhile, the cells delivered with both miR-424 inhibitor and sh-MYB exhibited no pronounced differences in cell proliferation. Transwell assay was then performed to detect the migration and invasion of SKOV-3 cells (Fig. 3d, e), and it was found that cells delivered with miR-424 mimic and with sh-MYB presented with repressed migration and invasion abilities, whereas the cells delivered with miR-424 inhibitor exhibited the opposite results (*p* < 0.05). However, there was no difference in migration and invasion abilities of cells delivered with both miR-424 inhibitor and sh-MYB. Lastly, Western blot analysis was conducted to detect the protein expression patterns of tube formation factors VEGF and VEGFR. The obtained results in Fig. 3f depicted that the protein expression of VEGF and VEGFR was both significantly suppressed after treatment with miR-424 mimic and with sh-MYB, however, an elevation in the protein expression of VEGF and VEGFR was observed following treatment with miR-424 inhibitor (*p* < 0.05). Overall, these results demonstrated that miR-424 suppressed the proliferation, migration, and invasion of ovarian cancer cells by inhibiting MYB, and miR-424 reduced the expressions of VEGF and VEGFR.

### The MSC-derived EVs are successfully isolated and identified

The effects of MSCs and MSC-derived EVs in various cancers have been well established, and the current study set out to expand on the role of MSC-derived EVs in ovarian cancer. Firstly, we isolated and seeded MSCs. After 3 days of culture, cells started to be adherent, presented with spindle-like shape and grew in a whirl-shape or cluster, while the nucleus was visible, overall, exhibiting the typical characteristics of MSCs (Additional file 3: Fig. S3A). Thereafter, flow cytometry was applied to analyze the expression patterns of MSC surface antigens, and the expressions of CD73 (91.56%), CD90 (88.93%), CD105 (86.55%), CD34 (21.57%), CD45 (19.63%), CD19 (25.48%), and HLA-DR (27.52%) were found in Additional file 3: Fig. S3B, which conformed to the biological characteristics of MSCs. Moreover, the results of MSCs osteogenic/adipogenic induction were depicted in Additional file 3: Fig. S3C. In addition, after 21 days of osteogenic induction, the cells were noted to be arranged in overlapping layers and presenting with calcified nodules containing a small amount of mineral salt deposition, highlighting the potential of osteogenesis differentiation in cells (Additional file 3: Fig. S3C-a). Moreover, after 25 days of adipogenic induction, significant lipid deposition was observed in the cells, while lipid droplets exhibited larger size and presented as beads-shape, which suggested the potential of adipogenesis differentiation of these cells (Additional file 3: Fig. S3C-b).

Furthermore, MSC-derived EVs were observed using TEM with the diameter of EVs measured by dynamic light scattering (DLS), and it was found that EVs (with size from 30 to 120 nm) presented a round or elliptic membranous vesicle-shapes (Additional file 3: Fig. S3D). Lastly, Western blot analysis was conducted to examine the expression patterns of CD63 and verified the successful extraction of EVs (*p* < 0.05) (Additional file 3: Fig. S3E). These results suggested the successful extraction of EVs from MSCs.

### miR-424 is delivered from MSCs to ovarian cancer cells via EVs

**Fig. 4:**
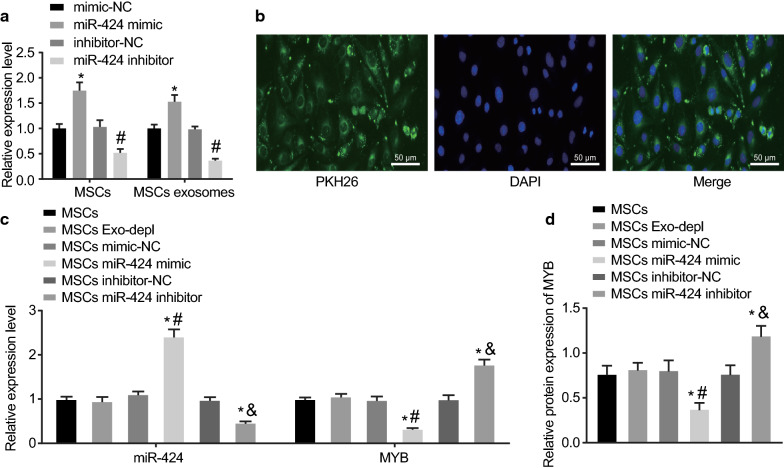
miR-424 is transferred from MSCs to ovarian cancer cells by EVs. **a** The expression of miR-424 in MSCs and MSC-derived EVs after treatments with miR-424 mimic and inhibitor as well as their NCs. **b** The uptake of EVs by ovarian cancer cells observed under a confocal fluorescence microscope (scale bar = 50 μm). The ovarian cancer cells SKOV-3 used for the following detections were co-cultured with MSCs, EVs-depleted MSCs, and MSCs treated with miR-424 mimic or miR-424 inhibitor as well as their NCs.** c** The expression of miR-424 and MYB in ovarian cancer cells detected by RT-qPCR. **d** The protein expression of MYB in ovarian cancer cells examined by Western blot analysis, with protein bands assessed. *p < 0.05 compared with MSCs, ^#^*p* < 0.05 compared with mimic NC and MSCs mimic NC treatments; & *p* < 0.05 compared with inhibitor NC and MSCs inhibitor NC treatments. The data above were measurement data and were presented as sample mean ± standard deviation. Data among multiple groups were compared by one-way ANOVA. The cell experiment was repeated 3 times independently

RT-qPCR was performed to detect the expression patterns of miR-424 in MSCs and MSC-derived EVs, and the results indicated that compared with MSCs treated with miR-NC, higher expression of miR-424 was noted in MSCs and MSC-derived EVs after treatment with miR-424 mimic, while lower miR-424 expression was found in MSCs and MSC-derived EVs after treatment with miR-424 inhibitor (*p* < 0.05) (Fig. 4a).

Subsequently, PKH67-labeled EVs were co-cultured with SKOV-3 cells for 48 h, and the uptake of EVs by SKOV-3 cells was assessed using confocal fluorescence microscopy. The results in Fig. 4b showed that after 48 h of co-culture, there was obvious uptake of PKH67-labeled EVs by SKOV-3 cells, suggesting that EVs could be internalized by ovarian cancer cells SKOV-3.

Furthermore, to determine the effective regulation of MYB expression by MSC-derived EVs carrying miR-424 in SKOV-3, RT-qPCR and Western blot analysis were conducted to examine the expression patterns of miR-424 and MYB in SKOV-3 co-cultured with MSC-derived EVs, respectively. As indicated in Fig. 4c, d, in comparison with MSCs, the MSCs treated with depleted EVs exhibited no significant differences in terms of MYB expression, while the MSCs treated with miR-424 mimic presented with significantly reduced expressions of MYB (*p* < 0.05), suggesting that miR-424 effectively suppressed the MYB expression in SKOV-3 cells in vitro. These results speculated the vital role of EVs transportation as MSCs transferred miR-424 to SKOV-3 cells through EVs.

### MSC-derived EVs over-expressing miR-424 suppresses the proliferation, migration and tube formation of HUVECs

**Fig. 5:**
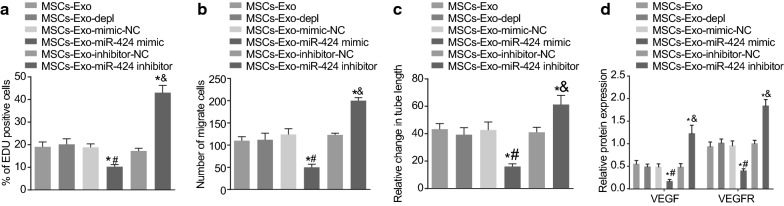
miR-424 from MSC-derived EVs inhibits the proliferation, migration and tube formation of HUVECs. The HUVECs used for the following detections were treated with MSC-derived EVs, EVs-depleted MSCs, MSC-derived EVs after treatments of miR-424 mimic or miR-424 inhibitor as well as their NCs. **a** The proliferation of HUVECs after different treatments detected by EdU assay. **b** The migration of HUVECs after different treatments examined by Transwell assay. **c** The tube formation of HUVECs after different treatments determined by Matrige-based tube formation assay. **d** The protein expression of VEGF and VEGFR after different treatments detected by Western blot analysis. *p < 0.05 compared with HUVECs treated with MSC-derived EVs, ^#^*p* < 0.05 compared with HUVECs treated with MSC-derived EVs-mimic-NC; ^&^*p* < 0.05 compared with HUVECs treated with MSC-derived EVs-inhibitor-NC. The data above were measurement data and were presented as sample mean ± standard deviation. Data among multiple groups were compared by one-way ANOVA. The cell experiment was repeated 3 times independently

HUVECs were treated with the supernatant of ovarian cancer cells co-cultured with MSC-derived EVs and followed by a series of experiments to detect the proliferation, migration, and tube formation of HUVECs. The results of the EdU assay (Fig. 5a) demonstrated that MSC-derived EVs-miR-424 mimic treatment-induced a pronounced decline in the proliferation of HUVECs, while MSC-derived EVs-miR-424 inhibitor treatment significantly enhanced the proliferation of HUVECs (*p* < 0.05). Meanwhile, the results of the Transwell assay (Fig. 5b) showed that migration of HUVECs was suppressed by MSC-derived EVs-miR-424 mimic treatment, while being promoted following treatment with MSC-derived EVs-miR-424 inhibitor (*p* < 0.05). Besides, the results of Matrigel-based tube formation assay (Fig. 5c) demonstrated the enhanced tube formation of HUVECs after treatment with MSC-derived EVs-miR-424 inhibitor (*p* < 0.05). Furthermore, Western blot analysis was conducted to examine the protein expression patterns of tube formation factors VEGF and VEGFR. As revealed in Fig. 5d, the protein expression of VEGF and VEGFR was found to be repressed by treatment with MSC-derived EVs-miR-4242 mimic, while the opposite results were noted after MSC-derived EVs-miR-424 inhibitor treatment (*p* < 0.05). These findings indicated that EVs-packaged miR-424 derived from MSCs repressed the proliferation, migration, and tube formation of HUVECs and inhibited the expression of VEGF and VEGFR.

### MSC-derived EVs over-expressing miR-424 inhibit the tumorigenesis and angiogenesis of ovarian cancer in vivo

**Fig. 6:**
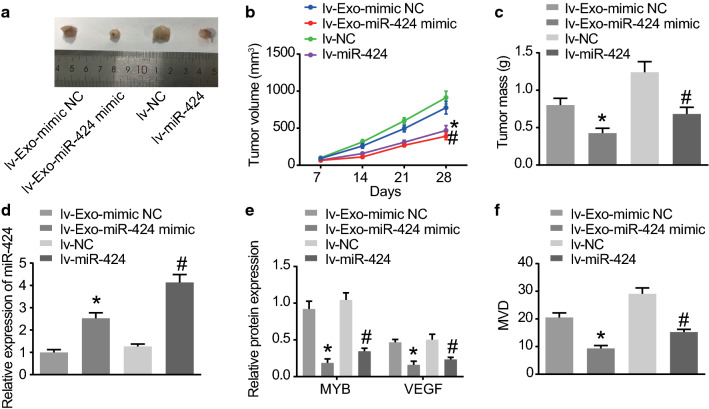
Overexpressed miR-424 delivered by MSC-derived EVs represses the tumorigenesis and angiogenesis of ovarian cancer in vivo. The nude mice used for the following detections were injected with cells transfected with lv-NC, lv-miR-424 mimic, EVs-mimic NC and EVs-miR-424 mimic. **a** The xenografted tumors from nude mice after different treatments. **b** The volume of tumors from nude mice after different treatments. **c** The weight of tumors from nude mice after different treatments. **d** The expression of miR-424 in EVs of serum from nude mice after different treatments, as detected by RT-qPCR. **e** The protein expression of MYB and VEGF in tumor tissues from nude mice after different treatments determined by Western blot analysis, with protein bands assessed. **f** The MVD of tumor tissues from nude mice after different treatments detected by MVD measurement. *p < 0.05 compared with lv-EVs-mimic NC treatment, ^#^*p* < 0.05 compared with lv-NC treatment. The data above were measurement data and were presented as sample mean ± standard deviation. Data among multiple groups were compared by one-way ANOVA, and the data of tumor volume were analyzed by repeated measurement ANOVA, n = 15

Lastly, modeled nude mice were intraperitoneally injected with MSC-derived EVs after different treatments and then with lentivirus-packaged plasmids, followed by euthanization after 2–4 weeks. Subsequently, the mice were dissected to obtain the xenografted tumors that were oval-shaped or irregular-shape, and all tumor tissues were confirmed by pathological analyses. The results showed that in comparison with lv-EVs-mimic NC treatment and lv-NC treatment, lv-EVs-miR-424 mimic treatment and lv-miR-424 treatment suppressed the tumorigenic ability (Fig. 6a), reduced the volume of tumors (Fig. 6b), and the weight of tumors (Fig. 6c) (all *p* < 0.05).

In addition, serum samples were obtained from nude mice after different treatments and analyzed with RT-qPCR to determine the miR-424 expression patterns. It was found that compared with lv-EVs-mimic NC treatment and lv-NC treatment, lv-EVs-miR-424 mimic treatment and lv-miR-424 treatment brought about increased expression of miR-424 (all *p* < 0.05) (Fig. 6d). Furthermore, Western blot analysis was employed to examine the protein expression patterns of MYB and VEGF in tumor tissues of nude mice after different treatments. As exhibited in Fig. 6e, the protein expression of MYB and VEGF was suppressed by miR-424 over-expression (*p* < 0.05). Finally, MVD measurement was used to examine the MVD of tumor tissues of nude mice after different treatments, with the obtained results indicating that the new blood vessels were brown yellow-stained or brown-stained cells or cell clusters. Moreover, compared to the treatments with lv-EVs-mimic NC and lv-NC, the treatments with lv-EVs-miR-424 mimic and lv-miR-424 led to remarkable decreases in MVD (Fig. 6f) (all *p* < 0.05). Collectively, these findings demonstrated that MSC-derived EVs over-expressing miR-424 hold the potential to suppress the tumorigenic ability of SKOV-3 cells and the MVD of tumor tissues.

## Discussion

Ovarian cancer is considered as the most prevalent and malignant tumor, due to late diagnosis and high relapse rate [23]. Interestingly, MSCs have emerged as potential therapeutic targets for various malignancies including ovarian cancer [24]. In addition to conferring an anti-tumor role in human epithelial ovarian cancer [25], human endometrial MSCs and/or their derived EVs have also been previously documented to exert suppressive effects on cancers [26]. Based on the obtained findings, we suggested that MSC-derived EVs overexpressing miR-424 suppressed the proliferation, migration, and invasion of ovarian cancer cells, thus repressing the tumorigenesis and angiogenesis of ovarian cancer.

Our initial findings revealed that MYB was highly-expressed and miR-424 was poorly-expressed in ovarian cancer. In addition, we uncovered that miR-424, delivered into ovarian cancer cells carried out its functions through MSC-derived EVs. Similarly, a previous study attributed up-regulated c-MYB levels to the damaging effect on the survival of patient with ovarian cancer [27]. Moreover, high expression of c-MYB has also been deciphered to be positively-correlated with tumor differentiation, ascites, lymph node metastasis and FIGO in patients affected by epithelial ovarian cancer [28]. On the other hand, another study detected reduced expression of miR-424 in 87.09% (27/31) of epithelial ovarian cancer tissues relative to corresponding adjacent non-tumor tissues [29]. Also, poor expression of miR-424 has been documented in radioresistant cervical cancer [30]. Further in line with our findings, repressed miR-424-5p levels were previously indicated and associated with clinicopathological features of epithelial ovarian cancer sufferers [31]. In addition, accumulating studies have demonstrated the crucial role and the effects of miRNA-containing EVs in ovarian cancer. For instance, EVs-mediated delivery of miRNAs was revealed to serve as a tumor biomarker in epithelial ovarian cancer [32]. Macrophages-derived EVs transferred miRNAs including miR-29a-3p and miR-21-5p have also been reported to cause a Treg/Th17 cell imbalance in epithelial ovarian cancer [33]. Additionally, miRNA-containing EVs conferred chemo-resistance in ovarian cancer via regulating the Cav1/p-gp/M2-type macrophage axis [34].

Further mechanistic investigation in our study demonstrated that MYB was a target gene of miR-424. Consistently, it has been reported that miR-424 targeted c-MYB, and consequently, repressed the invasion and migration of hepatocellular carcinoma cells via c-MYB [35]. Other studies have further stated that regulation of VEGF production by MYB has implications for the potential role of MYB in myeloid leukemia and solid tumors, wherein VEGF may act as an autocrine growth factor [36]. The transcription factor B-MYB is a pVHL substrate that can be degraded by the ubiquitin–proteasome pathway and VEGF- and/or platelet-derived growth factor-dependent tyrosine 15 phosphorylation of B-MYB prevents its degradation [37]. In our study, we found that MSCs could deliver miR-424 into ovarian cancer cells to target MYB, which further inhibited the expression of VEGF and also endothelial cell proliferation, migration as well as tube formation, thereby suppressing the angiogenesis of ovarian cancer.

Additionally, subsequent findings in our study indicated that the MSC-derived EVs over-expressing miR-424 could significantly repress the proliferation, migration, and tube formation of HUVECs by constraining MYB, which further led to the inhibition of the tumorigenesis and angiogenesis of ovarian cancer. In accordance with our findings, miR-424 was previously illustrated to be poorly-expressed in ovarian clear cell carcinoma, and further exhibited an inhibitory role on malignant phenotypes in ovarian clear cell carcinoma via doublecortin-like kinase 1 suppression [12]. The inhibitory role of miR-424-5p on proliferation and migration of ovarian cancer cells has been previously established [11]. Moreover, the down-regulation of miR-424 has been shown to aggravate the abnormal angiogenesis and cell proliferation in senile hemangioma [38], which again underscores the effect of miR-424 overexpression on repressing tumor angiogenesis. Furthermore, existing evidence highlights the involvement of miRNA-containing EVs in the angiogenesis of ovarian cancer by indicating the contribution of miR-141-3p-carrying EVs in the angiogenesis of endothelial cells in ovarian cancer via the activation of the JAK/STAT3 and NF-κB pathway [39]. Nonetheless, a comprehensive understanding of the role of miR-424-containing EVs in ovarian cancer is a prerequisite to further translate these experimental findings into clinical trials to improve the quality of life of patients plagued by ovarian cancer.

## Conclusion

**Fig. 7:**
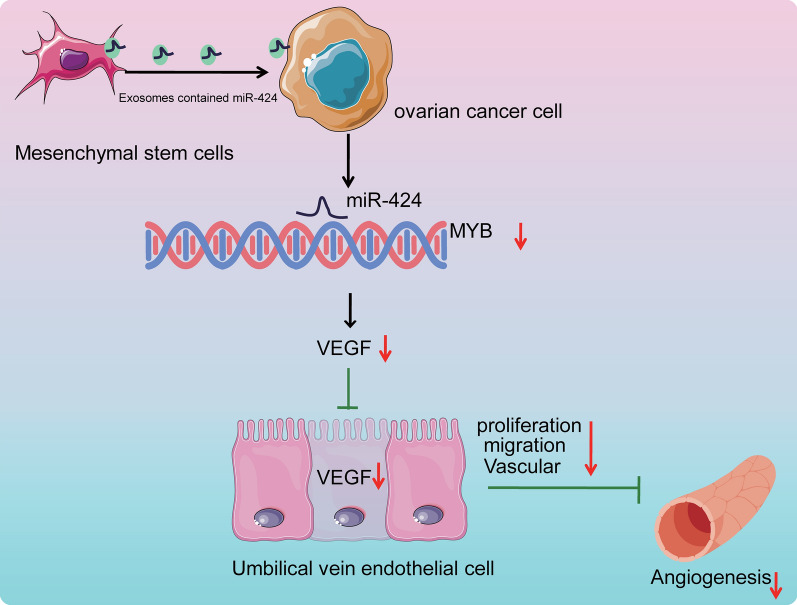
The molecular mechanism of MSC-derived EVs carrying miR-424 in affecting ovarian cancer. miR-424 is delivered from MSCs to ovarian cancer cells via EVs. miR-424 targets and inhibits MYB expression. MSC-derived EVs carrying miR-424 repress the expression of VEGF, thus inhibiting the proliferation, migration and tube formation of HUVECs and further suppressing the tumorigenesis and angiogenesis of ovarian cancer

Collectively, findings obtained in the current study demonstrated that EVs derived from MSCs shuttled over-expressed miR-424 into ovarian cancer cells to influence the development of ovarian cancer. Additionally, we illustrated that MSCs secreted miR-424-containing EVs, which suppressed the proliferation, migration, and tube formation of HUVECs via MYB inhibition and ultimately, attenuated the tumorigenesis and angiogenesis of ovarian cancer (Fig. 7). Therefore, it would be plausible to suggest MSC-derived EVs enriched with over-expressed miR-424 as a promising candidate for developing novel therapeutics for ovarian cancer. Our research is at the preclinical stage, and thus further investigation is warranted to elucidate the underlying mechanism of MSCs secreted miR-424-containing EVs in ovarian cancer and develop them into clinical trials.

## Supplementary Information

**Additional file 1:** **Figure S1.** Agarose gel electrophoresis of EVs-RNA.

**Additional file 2: Figure S2.** Differential analysis of ovarian cancer gene datasets GSE18520 (**a**) and GSE38666 (**b**).

**Additional file 3:** **Figure S3.** EVs are successfully isolated from MSCs.

## Data Availability

The authors confirm that the data supporting the findings of this study are available within the article.

## Abbreviations

EVs: Extracellular vesicles
MSC: Mesenchymal stem cell
miR-424: microRNA-424
BDs: Binding domains
GEO: Gene Expression Omnibus
DEGs: Differentially expressed genes
PPI: Protein-protein interaction
cDNA: Complementary DNA
GAPDH: Glyceraldehyde-3-phosphate dehydrogenase
